# Systematic review of the Hawthorne effect: New concepts are needed to study research participation effects^☆^

**DOI:** 10.1016/j.jclinepi.2013.08.015

**Published:** 2014-03

**Authors:** Jim McCambridge, John Witton, Diana R. Elbourne

**Affiliations:** a Department of Social & Environmental Health, Faculty of Public Health and Policy, London School of Hygiene & Tropical Medicine, 15-17 Tavistock Place, London WC1H 9SH, UK; b National Addiction Centre, Institute of Psychiatry, King's College London, 4 Windsor Walk, London SE5 8AF, UK; c Department of Medical Statistics, Faculty of Epidemiology and Population Health, London School of Hygiene & Tropical Medicine, Keppel Street, London WC1E 7HT, UK

**Keywords:** Hawthorne effect, Reactivity, Observation, Research methods, Research participation, Assessment

## Abstract

**Objectives:**

This study aims to (1) elucidate whether the Hawthorne effect exists, (2) explore under what conditions, and (3) estimate the size of any such effect.

**Study Design and Setting:**

This systematic review summarizes and evaluates the strength of available evidence on the Hawthorne effect. An inclusive definition of any form of research artifact on behavior using this label, and without cointerventions, was adopted.

**Results:**

Nineteen purposively designed studies were included, providing quantitative data on the size of the effect in eight randomized controlled trials, five quasiexperimental studies, and six observational evaluations of reporting on one's behavior by answering questions or being directly observed and being aware of being studied. Although all but one study was undertaken within health sciences, study methods, contexts, and findings were highly heterogeneous. Most studies reported some evidence of an effect, although significant biases are judged likely because of the complexity of the evaluation object.

**Conclusion:**

Consequences of research participation for behaviors being investigated do exist, although little can be securely known about the conditions under which they operate, their mechanisms of effects, or their magnitudes. New concepts are needed to guide empirical studies.

## 1 Introduction

**What is new?**

• Most of the 19 purposively designed evaluation studies included in this systematic review provide some evidence of research participation effects.
• The heterogeneity of these studies means that little can be confidently inferred about the size of these effects, the conditions under which they operate, or their mechanisms.
• There is a clear need to rectify the limited development of study of the issues represented by the Hawthorne effect as they indicate potential for profound biases.
• As the Hawthorne effect construct has not successfully led to important research advances in this area over a period of 60 years, new concepts are needed to guide empirical studies.

The Hawthorne effect concerns research participation, the consequent awareness of being studied, and possible impact on behavior [1-5]. It is a widely used research term. The original studies that gave rise to the Hawthorne effect were undertaken at Western Electric telephone manufacturing factory at Hawthorne, near Chicago, between 1924 and 1933 [6-8]. Increases in productivity were observed among a selected group of workers who were supervised intensively by managers under the auspices of a research program. The term was first used in an influential methodology textbook in 1953 [9]. A large literature and repeated controversies have evolved over many decades as to the nature of the Hawthorne effect [5,10]. If there is a Hawthorne effect, studies could be biased in ways that we do not understand well, with profound implications for research [11].

Empirical data on the Hawthorne effect have not previously been evaluated in a systematic review. Early reviews examined a body of literature on studies of school children and found no evidence of a Hawthorne effect as the term had been used in that literature [12-14]. The contemporary relevance of the Hawthorne effect is clearer within health sciences, in which recent years have seen an upsurge in applications of this construct in relation to a range of methodological phenomena (see examples of studies with nonbehavioral outcomes [15-17]).

There are two main ways in which the construct of the Hawthorne effect has previously been used in the research literature. First, there are studies that purport to explain some aspect of the findings of the original Hawthorne studies. These studies involve secondary quantitative data analyses [1,18-20] or discussions of the Hawthorne effect, which offer interpretations based on other material [4,10,21,22]. The Hawthorne effect has also been widely used without any necessary connection to the original studies and has usually taken on the meaning of alteration in behavior as a consequence of its observation or other study. In contrast to uses of the term in relation to the original Hawthorne studies, methodological versions of the Hawthorne effect have mutated in meaning over time and across disciplines and been the subject of much controversy [1,2,4,23,24]. This diversity means that certain aspects of the putative Hawthorne effect, for example, novelty [25] are emphasized in some studies and are absent in many others.

There is a widespread social psychological explanation of the possible mechanism for the Hawthorne effect as follows. Awareness of being observed or having behavior assessed engenders beliefs about researcher expectations. Conformity and social desirability considerations then lead behavior to change in line with these expectations. Chiesa and Hobbs [5] point out that just as there are different meanings given to the purported Hawthorne effect, there are also many suggested mechanisms producing the effect, some of which are contradictory. In all likelihood, the most common use of the Hawthorne effect term is as a post hoc interpretation of unexpected study findings, particularly where they are disappointing, for example, when there are null findings in trials.

The aims of this systematic review were to elucidate whether the Hawthorne effect exists, explore under what conditions, and estimate the size of any such effect, by summarizing and evaluating the strength of evidence available in all scientific disciplines. Meeting these study aims contributes to an overarching orientation to better understand whether research participation itself influences behavior. This inclusive orientation eschews restrictions on participants, study designs, and precise definitions of the content of Hawthorne effect manipulations.

## 2 Methods

The Hawthorne effect under investigation is any form of artifact or consequence of research participation on behavior.

Studies were included if they were based on empirical research comprising either primary or secondary data analyses; were published in English language peer-reviewed journals; were purposively designed to determine the presence of, or measure the size of, the Hawthorne effect, as stated in the introduction or methods sections of the article or before the presentation of findings if the report is not organized in this way; and reported quantitative data on the Hawthorne effect on a behavioral outcome either in observational designs comparing measures taken before and after a dedicated research manipulation or between groups in randomized or nonrandomized experimental studies. Behavioral outcomes incorporate direct measures of behavior and also the consequences of specific behaviors. Studies that described their aims in other ways and also referred to the Hawthorne effect as an alternative conceptualization of the object of evaluation were included as were studies that have other primary aims such as the evaluation of an intervention in a trial in which assessment of the Hawthorne effect is clearly stated as a secondary aim of the study, for example, with the incorporation of control groups with and without Hawthorne effect characteristics. Studies were excluded if: unpublished or in grey literature on the grounds that it is not possible to systematically assess these literature in an unbiased manner; discussion articles and commentaries were not considered to constitute empirical research; they referenced or used the term Hawthorne effect incidentally or described it as a design feature or as part of the study context, or invoked it as an explanation for study findings. Studies of the Hawthorne effect that incorporate nonresearch components, including cointerventions such as feedback, hamper evaluation and are also excluded, as were reanalyses of the original Hawthorne factory data set by virtue of nonresearch cointerventions such as managerial changes (see Ref. [8] for a detailed history of the studies).

Studies were primarily identified in electronic databases. In addition, included studies and key excluded references were backward searched for additional references and forward searched to identify reports that cited these articles. Experts identified in included studies and elsewhere were contacted. The most recent database searches took place on January 3, 2012 for the following databases: Web of Science (1970-), MEDLINE (1950-), BIOSIS Previews (1969-), PsycInfo (1806-), CINAHL Plus with full text (1937-), ERIC (1966-), PubMed (1950-), Cochrane Central Register of Controlled Trials (1947-), Embase (1947-), Sociological Abstracts (1952-), National Criminal Justice Reference Service Abstracts (NCJRS; 1970-), Social Services Abstracts (1979-), Linguistics and Language Behavior Abstracts (LLBA; 1973-), the International Bibliography of the Social Sciences (1951-), APPI Journals (1844-), British Nursing Index (1992-), ADOLEC (1980-), Social Policy and Practice (1890-), British Humanities Index (1962-), Applied Social Sciences Index and Abstract (1987-), Inspec (1969-), and PsycARTICLES (1988-).

The term "Hawthorne effect" was searched for as a phrase as widely as possible. If the database permitted, the term was searched for in all fields as was the case for Embase, CINAHL, ERIC, and others. In the Web of Knowledge search, which uses Web of Science, MEDLINE, and BIOSIS Previews databases, "Hawthorne effect" was entered into the "topic" field. The term was also searched for in "keyword" fields for databases such as NCJRS, LLBA, APPI, and others. The use of this term as the core object of evaluation negated the need for a more complex search strategy.

Hits from the database searches were downloaded into EndNote software (Thomson Reuters), removing duplicates there. Screening of titles and abstracts was undertaken by the second author or a research assistant. After a further brief screen of full-text articles, potential inclusions were independently assessed before being included. Data extracted are summarized in the tables presented here, which also contain information on risk of bias in individual studies. Binary outcomes were meta-analyzed in Stata, version 12 (Statacorp), with outcomes pooled in random-effects models using the method by DerSimonian and Laird. The *Q* and *I*^2^ statistics [26] were used to evaluate the extent and effects of heterogeneity, and outcomes are stratified by study design. Formal methods were not used to assess risk of bias within and across studies, and narrative consideration is given to both. We did not publish a protocol for this review.

## 3 Results

**Fig. 1:**
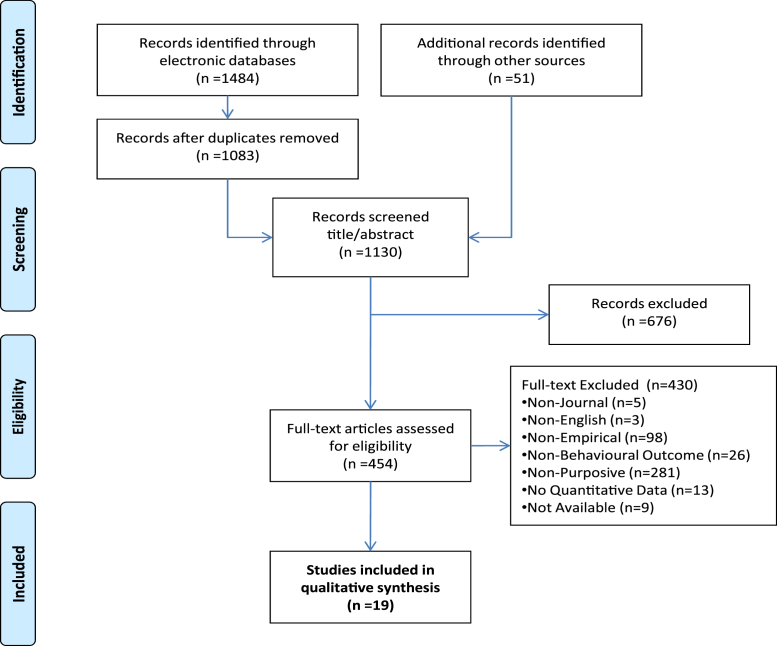
PRISMA flowchart.

Nineteen studies were eligible for inclusion in this review [27-45]. The PRISMA flowchart summarizing the data collection process is presented in Fig. 1. The design characteristics of included studies, along with brief summaries of outcome data and observations on most likely sources of bias, are presented separately for randomized controlled trials (RCTs), quasiexperimental studies, and observational studies in Tables 1-3, respectively. All included studies apart from one [27] have been undertaken within health sciences. All observational studies were studies of the Hawthorne effect on health-care practitioners, as were two of the quasiexperimental studies [37,39]. Although none of the randomized trials evaluate possible effects on health-care practitioners, the study by Van Rooyen et al. [28] was undertaken with health researchers. The quasiexperimental studies tend to have been conducted before both the RCTs and observational studies, for which the clear majority of both types of studies have been reported within the past decade. The oldest included study was published approximately 25 years ago [35]. Four of the 5 quasiexperimental studies used some form of quasirandomized methods in constructing control groups (except Ref. [36]). Heterogeneity in operationalization of the Hawthorne effect for dedicated evaluations, in study populations, settings, and in other ways, is readily apparent in Tables 1-3.

**Fig. 2:**
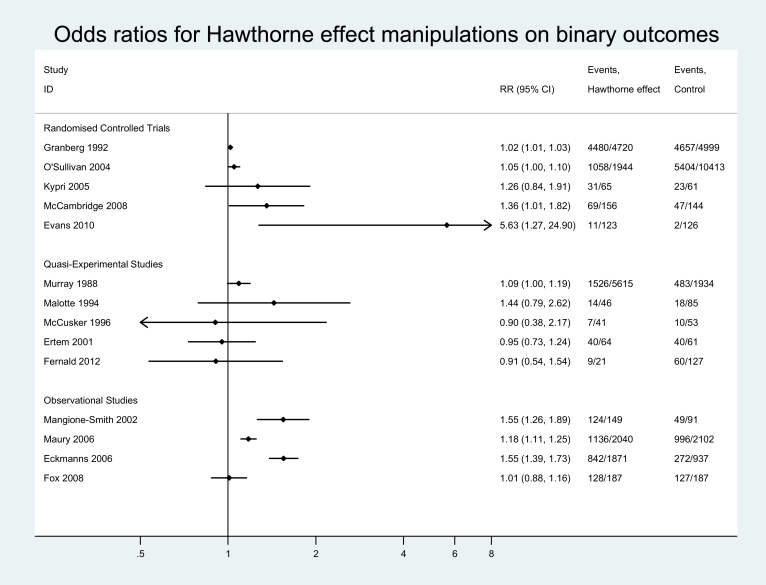
Binary outcome data. RR, relative risk; CI, confidence interval.

Fourteen of the 19 included studies report evaluations of effects on binary outcome measures. These data are presented in Fig. 2. The first six studies presented in Fig. 2 comprise six of the seven (not including Ref. [32]) evaluations of the effects of reporting on one's behavior by answering questions either in interviews or by completing questionnaires. All other studies evaluate being directly observed and/or the awareness of being studied in various ways, apart from one study that combines both types of Hawthorne effect manipulation [33].

As a result of heterogeneity in definitions of the Hawthorne effect (reflecting the inclusion criteria), findings from meta-analytic syntheses should be treated with caution. Explorations of the extent and effects of heterogeneity are presented in Table 4. Pronounced effects of statistical heterogeneity are reflected in the *I*^2^ statistics for two of the three study designs (RCTs and observational studies) and also when attention is restricted to the eight studies of being observed or studied and to the subset of six studies of answering questions, and overall. When the one interview study (of preelection interview effects on voter turnout [27]) is removed, however, to leave five studies of the effects of self-completing questionnaires on health behaviors, statistical heterogeneity is markedly attenuated.

Bearing these explorations of heterogeneity in mind, effect estimates provide a confidence interval (CI) including unity for the five trials alone [odds ratio (OR), 1.06; 95% CI: 0.98, 1.14], and for the six studies of answering questions (OR, 1.07; 95% CI: 1.00, 1.15). They reach statistical significance in relation to the five studies of self-completing health questionnaires (OR, 1.11; 95% CI: 1.0, 1.23). The pooled estimate for the five quasiexperimental studies is similar to that for the five trials and is not statistically significant (OR, 1.07; 95% CI: 0.99, 1.17), whereas that for the four observational studies (OR, 1.29; 95% CI: 1.06, 1.30) and the eight studies of being observed (OR, 1.21; 95% CI: 1.03, 1.41) are larger and statistically significant. The overall odds ratio, without any weighting for study design, was 1.17 (95% CI: 1.06, 1.30).

Quantitative outcome data were presented in three of the other five studies; two identifying between-group differences [29,32] and one not [28]. The large effect in the study by Feil et al. [29] is noteworthy. In the remaining two studies, continuous measures of effect were not reported in the form of mean differences and were complex to interpret, although both reported statistically significant Hawthorne effect findings [40,43]. Continuous outcomes were also evaluated for two studies included in Fig. 1, with both finding evidence of statistically significant effects [33,35].

Of the 19 studies, therefore, 12 provided at least some evidence of the existence of a Hawthorne effect, however this was defined, to a statistically significant degree. Small sample sizes appeared to preclude between-group differences reaching statistical significance in two studies [31,36]. In five studies, it was judged clear that there were no between-group differences that could represent a possible Hawthorne effect [28,37-39,45].

## 4 Discussion

The Hawthorne effect has been operationalized for study as the effects of reporting on one's behavior by answering questions, being directly observed, or otherwise made aware of being studied. There is evidence of effects across these studies, and inconsistencies in this evidence. We explore heterogeneity in targets for, and methods of, study as well as in findings. We will begin by examining the evidence base for each of the study designs, before considering the limitations, interpretation, and implications of this study.

The RCTs tend to provide evidence of small statistically significant effects. There are also studies that showed no effects, and two studies that provided evidence of large effects [29,34]. The study by Feil et al. [29] used a strong manipulation, incorporating a placebo effect, which is not usually considered to be a Hawthorne effect component, in addition to research- and trial-specific participation effects. In both this study and the one undertaken by Evans et al. [34] also finding a large effect, small numbers of participants are involved. The diversity in the content of the manipulations in these studies is emphasized. When one considers the RCT data from the five studies contributing to the meta-analysis [27,30,31,33,34] alongside the three studies that did not, two of which produce statistically significant effects on continuous outcomes [29,32], it seems that overall, there is evidence of between-group differences in the RCTs. These between-group differences cannot, however, be interpreted to provide consistent or coherent evidence of a single effect.

The same could be said of the diversity of the contents of the manipulations in the quasiexperimental studies, and the picture is made more complex by greater variability in study design features, particularly in relation to allocation methods. Overall, they produce mixed evidence, with a between-group difference in one study [35], a noteworthy difference in a small underpowered study [36], and no evidence of between-group differences in the other three studies [37-39]. It is difficult to draw any conclusion from included studies with quasiexperimental designs.

Although their design precludes strong conclusions because of the likelihood of unknown and uncontrolled biases, the heterogeneity of findings in the observational studies is interesting. Two studies produce identical point estimates of effects [41,42], whereas the other two estimates are similarly different [44,45]. These data suggest that the size of any effects of health-care practitioners being observed or being aware of being studied probably very much depends on what exactly they are doing. Perhaps, this is not at all surprising, although it does undermine further the idea that there is a single effect, which can be called the Hawthorne effect. Rather, the effect, if it exists, is highly contingent on task and context. It is noteworthy that the three other studies with health professionals, all using control groups, show no effects [28,37,39].

This is an "apples and oranges" review. This approach was judged appropriate, given the current level of understanding of the phenomena under investigation. This design decision does, however, entail limitations in the forms of important differences between studies, including operationalization of the Hawthorne effect, and exposure to varying forms of bias. The observed effects are short term when a follow-up study is involved, with only the studies by Murray et al. [35] and Kypri et al. [32] demonstrating effects beyond 6 months. Both these studies involved repeated prior assessments, and both provide self-reported outcome data. Self-reported outcomes do not appear obviously more likely than objectively ascertained outcomes to show effects. The forms of blinding used are often tailored to the nature of the study, making performance bias prevention difficult to evaluate across the studies as a whole.

By design, we have excluded studies that defined the object of evaluation to incorporate nonresearch elements as occurred in the original studies at Hawthorne [8]. We may have missed studies that should have been identified, although this is unlikely if use of the Hawthorne effect term was in any way prominent. Studies that have been missed may be more likely to be older and from nonhealth literature. An alternative design for this study might have eschewed this label and sought instead to synthesize findings on studies of research participation and/or awareness of being studied. Although potentially attractive, this course of action would have involved considerable difficulties in identifying relevant material and would risk losing the main focus on the Hawthorne effect. Similarly, we might have also included studies with cognitive and/or emotional outcomes in which effects might be greater [46], rather than focusing on behavioral outcomes. This possibility may be appropriate for evaluation in the future. Although we have sought to make our explorations of the heterogeneity of included studies as informative as possible, our analyses might be seen as excessive data fishing. These are, however, clearly presented as post hoc analyses after examination of high levels of heterogeneity, and the study by Granberg and Holmberg [27] is distinct from the other four questionnaire studies in a range of ways including the behavior being investigated.

Heterogeneity in operationalization of the Hawthorne effect make the data in this review challenging to interpret, yet it does appear that research participation can and does influence behavior, at least in some circumstances. The content and strength of the Hawthorne effect manipulations vary in these primary studies, and so to do the effects, although it would not be possible to discern any form of dose-response relationship. The manipulation by Evans et al. [34] may appear in some respects weak, for example, being an online questionnaire, and in others as potentially strong, examining decision making in relation to uptake of a cancer test, which was the study outcome. Although weak uses of the Hawthorne effect term in the wider literature mean that it is not very informative for interpreting the data from this study, outcomes may be considered in relation to the prevailing ideas about the core mechanism of the Hawthorne effect that conformity to perceived norms or researcher expectations drives change. Many, but not all, of the studies with positive findings appear broadly consistent with this account, although so too do many of the studies with negative findings and it is not clear why this is so. The study by Murray et al. [35] examined adolescent smoking at a time when the prevalences of both nonsmoking and regular smoking were approximately one-quarter in this sample, so it is unclear what norms or perceptions of researcher expectations many have been. This study exemplifies the literature as a whole in being principally concerned with the possible existence of a Hawthorne effect and not being designed to test the hypothesized mechanism.

There are other possible mechanisms of effect that have also not been evaluated. For example, regardless of perceptions of norms or researcher expectations, the content of the questions asked may themselves stimulate new thinking. In the studies by Evans et al. [34] and O'Sullivan et al. [30], patients may well not have previously considered what was being enquired about, and this may have been an independent source of change. Concerns about biases being introduced by having research participants complete questionnaires have existed for more than 100 years, before the Hawthorne factory studies took place and approximately 50 years before the Hawthorne effect term was introduced to the literature (see Ref. [47] for an early history of these issues). Given how long the Hawthorne effect construct has been the predominant conceptualization of these phenomena [48], it appears that this construct is an inadequate vehicle for advancing understanding of these issues. Alternative long-standing conceptualizations of these problems such as demand characteristics within psychology have also yielded disappointingly underdeveloped research literatures [49-51]. This state of affairs points toward an obvious need for further study of whether, when, how, how much, and for whom research participation may impact on behavior or other study outcomes.

Further studies will be assisted by the development of a conceptual framework that elaborates possible mechanisms of effects and thus targets for study. The Hawthorne effect label has probably stuck for so long simply because we have not advanced our understanding of the issues it represents. We suggest that unqualified use of the term should be abandoned. Specification of the research issues being investigated or described is paramount, regardless of whether the Hawthorne label is seen to be useful or to apply or not in any particular research context. Perhaps, use of the label should be restricted to evaluations in which conformity and social desirability considerations are involved, although it is striking how hostile social psychology has been to this construct [2]. So, what can be said about priority targets for further study on the basis of this systematic review and what concepts are available to guide further study?

Decisions to take part in research studies may also be implicated in efforts to address behavior in other ways so that research participation interacts with other forces influencing behavior. It is also possible, if not likely, that these relatively well-studied types of data collection (completing questionnaires and being observed) are part of a series of events that occur for participants in research studies that have potential to shape their behavior, from recruitment onwards. Giving attention to precisely what we invite research participants to do in any given study seems a logical precursor to examination of whether any aspect of taking part may influence them. Phenomenological studies, which ask participants about their experiences, would seem to be useful for developing new concepts. If individual study contexts are indeed important, we should expect to see effects that vary in size and across populations and research contexts, and perhaps also with multiple mechanisms of effects. The underdeveloped nature of these types of research questions means that it may be unwise to articulate advanced conceptual frameworks to guide empirical study. We propose "research participation effects" as a starting point for this type of thinking. Although descriptive, it also invites investigation of other aspects of the research process beyond data collection, which may simply be where research artifacts emanating from both social norms and other sources are most obvious.

We conclude that there is no single Hawthorne effect. Consequences of research participation for behaviors being investigated have been found to exist in most studies included within this review, although little can be securely known about the conditions under which they operate, their mechanisms of effects, or their magnitudes. Further research on this subject should be a priority for the health sciences, in which we might expect change induced by research participation to be in the direction of better health and thus likely to be confounded with the outcomes being studied. It is also important for other domains of research on human behavior to rectify the limited development of understanding of the issues represented by the Hawthorne effect as they suggest the possibility of profound biases.

## Figures and Tables

**Table 1:** Study characteristics and findings of randomized controlled trials evaluating the Hawthorne effect

Characteristic	Granberg and Holmberg [27]	Van Rooyen et al. [28]	Feil et al. [29]	O'Sullivan et al. [30]	Kypri et al. [31]	Kypri et al. [32]	McCambridge and Day [33]	Evans et al. [34]
Population	Swedish general population	Academic peer reviewers	Adolescent dental patients	Colorectal cancer screening population	University students	University students	University students	Male prostate cancer-testing population
Setting	Community	Correspondence	Dental clinic	Community	Student health service	Student health service	Student union	General practice initiated internet
Operationalization of HE	Being interviewed before an election	Awareness of study participation	Participation in experimental arm of clinical trial	Being given a questionnaire with screening kit	Completing a questionnaire	Completing a questionnaire	Completing a questionnaire on alcohol	Completing a questionnaire
Comparison group	Being interviewed after an election	No awareness of study participation	Usual care	No questionnaire with screening kit	No questionnaire	No questionnaire	No questionnaire on alcohol	No questionnaire
Participant blinding	Not clear if aware of outcome assessment	Yes. Control group blinded to study conduct	Yes. HE group blinded to study purpose. Control group blinded to all aspects of study participation.	Not clear if aware of outcome assessment	Yes. Both groups blinded to conduct of trial and study purpose	Yes. Both groups blinded to conduct of trial and study purpose.	Yes. Both groups blinded to study purpose and focus on drinking, HE group capable of inferring the latter	Not clear if aware of outcome assessment
Outcome measure	Objectively ascertained voting records	Quality of reviews produced.	Objectively ascertained plaque scores	Uptake of screening ascertained in records	4 Self-reported health behaviors	Self-reported drinking and related problems	Self-reported drinking and related problems	Uptake of prostate cancer test in medical records
Follow-up intervals	Not reported	Not reported	3 and 6 mo	6 wk and 6 mo	6 wk	6 and 12 mo	2-3 mo	6 mo
Sample size	Preelection interview: 4,720, postelection interview: 4,999	316 unaware, 149 aware	40, 20 per group	1,944 sent a questionnaire, 10,413 not sent one	74 completed a questionnaire, 72 did not	147 completed a questionnaire, 146 did not	217 completed a questionnaire, 204 did not	150 per group
Attrition	None	None	Two lost to follow-up (one in each group)	None	86%, Not differential by group	84% and 86% not differential by group	77%, Not differential by group	83%, Not differential by group
Summary of reported findings	People interviewed before the election were more likely to vote (95% vs. 93%), and this effect was stronger for those with low political interest (93% vs. 90%)	No evidence of any difference	Large between-group differences in plaque score at both 3 (54 ± 13.79 vs. 78 ± 12.18) and 6 mo (52 ± 13.04 vs. 79 ± 10.76)	Small statistically significant differences in uptake at 6 wk (54.4% vs. 51.9%), no longer significant at 6 mo (64.7% vs. 62.9%)	No differences detected	No differences detected at 6 mo, 3 of 7 statistically significant differences in outcomes at 12 mo	Small statistically significant differences in 4 of 9 outcomes including primary outcome (0.23 standard deviations)	Those completing questionnaire more likely to undergo test (11 of 123 vs. 2 of 126)
Reviewer comments including on principal risks of bias	Completed interviews analyzed, not ITT. Various other sample refinements. Not a formal research report, details of study design and methods unclear.	Validated outcome measure completed by author. Potential for information bias unclear.	Family of three randomized ad hoc as cluster. Small study.	Sequence generation for "every sixth person" not described. Effects of reminder letters not reported.	Self-report.	Self-report. Reasons for lack of effect at 6 mo unclear.	Self-report. Attrition.	Two of four arms in online trial evaluating a decision support aid described. Limited information in report. Small numbers.

*Abbreviation*: HE, Hawthorne effect.

**Table 2:** Study characteristics and findings of quasiexperimental evaluations of the Hawthorne effect

Characteristic	Murray et al. [35]	Malotte and Morisky [36]	McCusker et al. [37]	Ertem et al. [38]	Fernald et al. [39]
Population	Secondary school children	Nonactive tuberculosis patients	General practitioners	Breast-feeding mothers of new born children	General practitioners
Setting	School	Tuberculosis clinic	General practice	Hospital	General practice
Operationalization of HE	Completing five annual questionnaires in cohort study	Participation in a usual care control arm of clinical trial	Practitioner completing a questionnaire on older patients' mental health	Participation in a cohort study	Completion of case reviews with researchers
Comparison group	Completing questionnaire only in the final year	Usual care group not participating in trial	No questionnaire	Eligible nonparticipants	No case reviews
Allocation method	Schools randomly selected at different times	Comparison group formed of all patients after monthly trial recruitment quota is reached	Alternate patient numbers, clinician-level data not reported	Alternate recruitment days	Random sample of 25% invited to participate.
Participant blinding	Yes to HE study purpose, not to focus on smoking behavior	HE group aware of trial participation, comparison group unaware of study	No	HE group aware of the cohort study of behavior. Comparison group unaware of the study	Not clear
Outcome measure	Self-reported smoking	Treatment retention	Recording	Data in routine records	Prescribing of antibiotics in abscess and cellulitis cases
Follow-up intervals	Cumulative exposure to 4 yr of surveys	6 and 12 mo	3 mo	2 wk, 2 and 4 mo	No follow-up. 6- to 7-mo study period
Sample size	5,615 annual questionnaires, 1,934 final year only, genders presented separately	46 in trial, 85 in nontrial group	41 with questionnaire, 53 without	64 in cohort study, 61 in nonparticipating group	91 clinicians, 14 participating in case reviews, and 77 comparisons
Attrition/response rates	75% in 48 HE schools, 84% in 12 of 20 control schools, differential between groups	No attrition, medical records used	No attrition, medical records used	No attrition, medical records used	No attrition, medical records used
Summary of reported findings	Two statistically significant differences in girls, one among boys, of four outcomes assessed: 23% vs. 29% nonsmokers among girls, 25% vs. 27% among boys. Outcomes aggregated for Fig. 1 here	At 6 mo, 30% vs. 21% comparing trial with nontrial groups, 12 mo 20% vs. 19%. Median time in treatment greater for trial group than nontrial group (13 vs. 5 wk). First follow-up data used in Fig. 1 here.	No differences in recording of mental health problems: 7 (17%) of 41 questionnaire; 10 (19%) of 53 no questionnaire.	No differences in discontinuation of breast-feeding: 2 wk 34% vs. 38%; 2 mo 73% vs. 70%; 4 mo 84% vs. 89% (nonparticipants vs. cohort study). First follow-up data used in Fig. 1.	No differences in antibiotic prescribing in reviewed abscess cases (9 of 21 vs. 60 of 127) or in reviewed cellulitis cases (105 of 250 vs. 465 of 1,108). First outcome data used in Fig. 1.
Reviewer comments including on principal risks of bias	Response rates differential at final survey. Between-group equivalence not demonstrated, vulnerable to selection bias.	Nonequivalent groups. Those not consenting to trial were excluded, no consent procedure for control group. Small sample size.	No data provided on clinicians. Unclear why imbalance in numbers allocated. May be weak manipulation of intended sense of being studied. Absence of blinding. Small study.	No consent procedure for control group, no information on refusals to consent to cohort study. Small sample size.	Outcome data reported comparing approximately 15% who participated in case reviews (rather than those randomized) with approximately 85% who did not. Brief report.

*Abbreviation*: HE, Hawthorne effect.

**Table 3:** Study characteristics and findings of observational study evaluations of the Hawthorne effect

Characteristic	Campbell et al. [40]	Mangione-Smith [41]	Eckmanns et al. [42]	Leonard and Masatu [43]	Maury et al. [44]	Fox et al. [45]
Population	Paramedics	Pediatricians	Clinicians	Clinicians	Clinicians	Obstetricians
Setting	Emergency services	Community practices	Hospital intensive care units	Outpatient clinics	Hospital intensive care unit	Hospital birth unit
Operationalization of HE	Announcement of study in a memo	Impact of audio taping consultations and completing questionnaires on inappropriate antibiotic prescribing	Announcement of 10-day direct observation study of hand hygiene	Direct observation of consultations by researchers	Announcement of observational study of hand hygiene in two time periods by two clinicians	Impact of awareness of being studied on diagnostic accuracy (EFW)
Comparisons	Prior awareness-raising memo	Unobserved consultations (neither audio/questionnaire) during same time period (and also later)	Covert observational period 10 mo earlier (same research nurse observer)	Nonobserved consultations before research team arrival	Two covert observed periods (by same two clinicians) when clinicians were unaware of being observed	Accuracy of estimates found in consecutive equivalent records in an earlier period
Blinding	No	Blinded to prescribing focus, consented to communication study	Only during covert observation	No	Only during covert observation	Were aware of study of accuracy, not of HE
Outcome measure	Documentation rates of medication, allergy, and medical history	Antibiotic prescribing in viral cases by direct observation or in medical records	Observed use of AHR	Patient-reported quality of care in postconsultation questionnaire	Observed hand hygiene compliance	Differences in accuracy (proportions of EFWs at birth weight ±10%)
Sample size	145 Practitioners and 30,828 reports	10 Pediatricians. 91 Nonobserved consultations, 149 observed	2,808 Indications for AHR use, 937 in period 1 and 1,871 in period 2	Not clear	4,142 Opportunities for hand hygiene compliance in four periods (1,064, 1,045, 1,038, and 995 each)	187 in each group
Summary of reported findings	Study led to increases in 2 of 3 recording outcomes (medication and allergy, not medical history). Regression coefficients and *P* values reported.	Inappropriate antibiotic prescribing in viral cases was 29% lower when observed (46% vs. 17%)	AHR compliance increased from 29% to 45% during the overt observation period (OR, 2.33; 95% CI: 1.95, 2.78; higher in multivariate model)	Patient-reported quality of care increased by 13% with direct observation, and returned to preobservation levels between 10 and 15 consultations	Both observed periods saw similar increases in compliance (47-55% and 48-56%).	No differences in main analysis (67.9% vs. 68.5% control/study). Possible difference in expected direction in small subgroup of heavy babies (37% vs. 53% control/study)
Reviewer comments including on principal risks of bias	No evaluation of confounding. Differences seen after awareness-raising memo, effect may be contingent.	Information bias judged likely. Reporting complex.	Big difference in indications for use in two periods reflects different observation procedures. Observer bias also possible.	Both the analyses and the reporting of outcome data are complex.	Not a formal report, so scant detail on data collection and other study procedures.	Equivalence problematic, clinicians different in two periods, although baby characteristics similar.

*Abbreviations*: HE, Hawthorne effect; EFW, estimate of fetal weight; AHR, antiseptic hand rub; OR, odds ratio; CI, confidence interval.

**Table 4:** Extent and effects of heterogeneity in 14 studies of the Hawthorne effect with binary outcomes^a^

	*Q*	df	*P*	*I*^2^ (%)
All 14 studies	194.47	13	<0.001	93.3
5 Randomized controlled trials	15.98	4	0.003	75.0
5 Quasiexperimental studies	2.35	4	0.67	0
4 Observational studies	32.63	3	<0.001	90.8
8 Studies of being observed or studied	38.25	7	<0.001	81.7
6 Studies of answering questions	23.23	5	<0.001	78.5
5 Studies of self-completing health questionnaires	8.9	4	0.064	55.0

a Rows 2-4 are mutually exclusive, as are rows 5 and 6. The final row 7 is a subset of data in row 6.
